# The University of Kansas Cancer Center (KUCC), an NCI designated comprehensive cancer center

**DOI:** 10.1186/s12943-023-01902-y

**Published:** 2023-11-28

**Authors:** Weijing Sun, Natalie Streeter, Joseph McGuirk, Roy Jensen

**Affiliations:** The University of Kansas Cancer Center, Kansas City, KS USA

**Keywords:** Comprehensive, Clinic research, Investigator-initiated trials


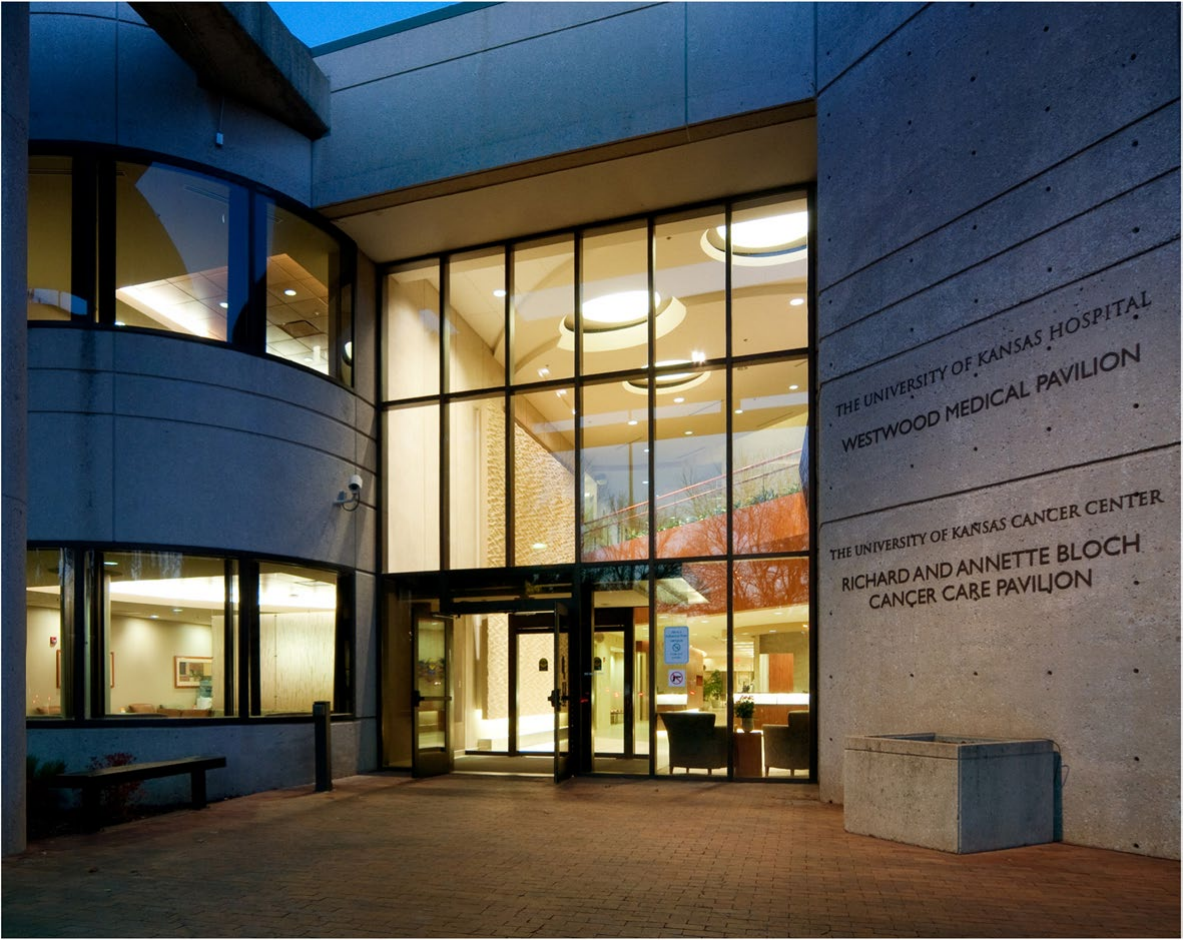


The University of Kansas Cancer Center is a National Cancer Institute-designated comprehensive cancer center. It is the only such designated cancer center in the state of Kansas. The cancer center’s mission, through its innovative approach to drug discovery, delivery, and development, is to transform cancer research and clinical care delivered in Kansas and western Missouri.

KU Cancer Center operates as a matrix organization that includes: the University of Kansas Medical Center campuses in Kansas City, Wichita and Salina, the University of Kansas in Lawrence and its highly ranked School of Pharmacy (per NIH funding) and The University of Kansas Health System. The Stowers Institute for Medical Research and Children’s Mercy Kansas City are NCI consortium partners.

KU Cancer Center places great emphasis on advancing discoveries in the laboratory to the patients’ bedside. The cancer center currently has five KU-invented anticancer agents in clinical trials. Two recent discoveries include Ciclopirox Prodrug for the treatment of bladder and other cancers, as well as a novel method to treat patients with acute Graft-versus-Host Disease.

The KU Cancer Center has garnered an excellent reputation for its clinical trials work and contributed to significant national studies and participates in exclusive NCI clinical trial network groups, including the ETCTN and NCORP. At any given time, the cancer center has nearly 600 studies in various stages.

KU Cancer Center researchers pay special attention to the unique needs of people living in its catchment area. Moreover, with the Children’s Mercy consortium partnership, KU Cancer Center has access to more than 95 percent of childhood cancer cases in the catchment area. The center’s outreach arm, the Masonic Cancer Alliance, leverages regional resources to promote and translate the latest evidence-based clinical and community health practices for patients close to their homes.

KU Cancer Center has three research programs that focus on different aspects of cancer and leverage scientific depth and breadth provided by nationally recognized scientists:

1. Cancer Biology, which aims to enhance interdisciplinary collaboration among basic and clinical scientists and facilitate translational research involving the etiology, treatment and prevention of cancer.
2. Cancer Prevention & Control, which seeks to identify new ways to prevent cancer and improve cancer outcomes, with a focus on high risk and underserved communities in KU Cancer Center’s catchment area.
3. Drug Discovery, Delivery & Experimental Therapeutics, which aims to discover novel anticancer drugs active against novel drug targets and find new indications for currently marketed drugs. The Institute for Advancing Medical Innovations (IAMI), which includes pharmaceutical industry experts, expands the program’s capacity to support drug discovery efforts across the entire Cancer Center.

## Clinic trials at The University of Kansas Cancer Center (KUCC)

With world-class, transdisciplinary, expertise and technical capabilities in drug discovery and cellular immunotherapeutics, spearheaded by our national and international leaders in translational and clinical research, KUCC continues to develop paradigm-changing therapeutic advances through leveraging multidisciplinary discoveries that drive translational research results into clinical trials which enable a comprehensive approach to improve cancer outcomes and quality of life and lead to comprehensive efforts locally and nationally to reduce the burden of cancer, particularly for people living in our extensive catchment area.

KUCC has a comprehensive clinical trial portfolio: including investigator-initiated trials (IIT) which emphasize KU-led novel scientific discovery/development, NCICTN national translational investigation trials, industry sponsored studies in multiple phases of development; as well as cooperative group, early concept proving phase I, II trials, many of which have the potential to lead to standard of care (SOC) changing phase III studies. Indeed, our collaboration with industry partners in new drug development and cellular therapeutic trials has led to the establishment of new clinical SOC and FDA approvals. In addition to our centers’ contributions into successful therapeutic clinical trials, we have trials employed to improve cancer prevention, and further the development and optimization of survivorship programs. Although KUCC clinical trials focus on patients' needs in the large KUCC catchment area, the results of our efforts have had far-reaching impacts nationally. Another high priority area of our research and clinical efforts at KUCC continues to be focused on defining disparities in access to cancer care and clinical trial enrollment for ethnic and racial, as well as socioeconomically disadvantaged populations in our catchment area. KUCC members have published extensively regarding these disparities and have multi-disciplinary efforts underway to positively impact these injustices. Since 2016 approximately 11–12% of KUCC new cancer patients have been enrolled to interventional clinical trials and, as a result, many of our patients have had the opportunity to contribute to advances in the field and potentially benefit from novel therapeutic strategies. It is our philosophy that clinical trials represent the best care for our patients, and our ultimate goal is that all patients who are referred to KUCC have available clinical trials for their consideration. Importantly, nearly 1/3 of our patients have participated in all cancer related studies including supportive interventional, supportive care, screening, service, diagnostic, and observational studies at KUCC. Approximately 120 clinical trials have opened, met enrollment goals, and subsequently closed each year (range 94–145) for the past 5 years with ~2,000 patients enrolled yearly (1,607–2,983). There are currently 327 open trials at KUCC including interventional trials composed of 36 phase I; 30 phase I/II; 79 phaseII; 12 phase II/III; 75 phase III: and 11 phase IV trials.

Several examples of studies which represent KUCC cancer clinic trials include the following:

An IIT of note involved a multi-PI Small Business Innovation Research grant supported by the NCI for the development of fosciclopirox for the treatment for high-risk non-muscle invasive bladder cancer. Bladder cancer is the fifth most common cancer in the United States, with over 83,000 new cases and over 17,000 deaths annually. In the KUCC catchment area, bladder cancer is the fourth most common cancer in men, and disproportionally impacts Black and Hispanic men. Discovered by KUCC scientists, fosciclopirox is a prodrug of ciclopirox, an FDA-approved topical antifungal agent with broad spectrum anticancer activity. Fosciclopirox is rapidly and completely converted to ciclopirox in the bloodstream where it subsequently undergoes renal elimination, selectively delivering ciclopirox to the entire urinary tract. KU investigators subsequently demonstrated in vitro and in vivo that ciclopirox was highly effective in killing human bladder cancer cells and established that intravenous administration of fosciclopirox at well tolerated doses in animals and humans results in urinary tract concentrations of ciclopirox which exceed those required to kill bladder cancer cells in vitro by > 50-fold. This KU-driven drug has been used in three phase 1 studies at KU and is also being researched in AML (Acute Myeloid Leukemia).

Another IIT that grew from KUCC-led science and multi-disciplinary collaboration is a triple negative breast cancer (TNBC) trial. TNBC is associated with a worse prognosis compared to other breast cancer subtypes. Using data from the KU multi-site TNBC registry, established in collaboration with the Biospecimen Shared Resource (BSR) and SWOG phase III trial S9313, several homologous recombination deficiency and immune microenvironment related chemotherapy response biomarkers were identified. This work led to the design and conduct of the NCI-funded SWOG phase II trial S1416 and ongoing multi-site IIT of neoadjuvant platinum chemotherapy plus immunotherapy in TNBC. Completed biomarker work demonstrated a relationship between immune infiltrate and treatment response and the ongoing work is interrogating interaction of tissue immune microenvironment/genomics and blood-based minimal residual disease markers with response to chemotherapy with or without immunotherapy. The KUCC supported AIM-TNBC IIT will assess the value of ctDNA based MRD for adjuvant treatment intensification in patients with high-risk TNBC. A Phase I/II IIT study of an oral, class I PI3K alpha-specific inhibitor (alpelisib) in combination with nab-paclitaxel chemotherapy in patients with metastatic HER2-negative breast cancer was conducted at KUCC and demonstrated very encouraging efficacy with an objective response rate of 52%. The clinical study was accompanied by pharmacokinetic studies performed by the Clinical Pharmacology Shared Resource and translational studies of tissue and ctDNA biomarkers supported by the Biospecimen Shared Resource. Based on the preliminary clinical/translational/PK data generated at KUCC, a Phase III registration trial of this regimen in biomarker selected patients with TNBC is ongoing. Further translational work is also underway to study novel ctDNA technologies for longitudinal MRD monitoring and assessment of resistance mechanisms for alpelisib. PI3K inhibitor, alpelisib, will be studied in combination with a novel antibody-drug conjugate in an upcoming Phase I study at KUCC. This highlight addresses breast cancer, a KUCC catchment area priority, and demonstrates transdisciplinary collaboration.

With the recognition of the broad anti-cancer efficacy of immune checkpoint inhibitors (ICI) in multiple advanced disease settings, a KUCC investigator pioneered a phase II study with combination chemotherapy and ICI in patients with adenocarcinoma of gastric, esophageal and gastro-esophageal junction (GEJ) in the peri-operative setting, which has the potential to lead to the standard of care management if efficacy is demonstrated by the phase III studies.

KUCC was among the very first in the United States to be certified as a CAR-T (chimeric antigen receptor T cell) center for Novartis, Kite/Gilead, and Bristol Myers Squibb and KUCC was the lead enrolling site to the pivotal Juliet trial which led to FDA approval for third line treatment of Diffuse Large B cell lymphoma (DLBCL). Additionally, KUCC center was a lead enrolling site to a pivotal, phase 3 prospective, randomized clinical trial comparing CAR-T therapy to autologous stem cell transplant for patients with relapsed or refractory DLBCL. This study demonstrated an EFS and OS (Overall Survival) advantage for CAR-T and led to FDA approval of CAR-T in the second line and changed the SOC in this clinical setting. Additionally, KUCC in partnership with the NCI and Children’s Mercy Hospital in Kansas City are pursuing a novel CAR-T construct that will target multiple different cell surface antigens using a single cell multi-antigen targeting CAR-T, thereby avoiding a common cause of treatment failure, i.e., antigen escape. Thus, KUCC has built and achieved national prominence for our CAR-T program. Further, KUCC holds several cell-therapy investigational new drug protocols (INDs) with the Food and Drug Administration and has initiated two corresponding investigator-initiated trials (IITs). The first IIT is investigating the safety and efficacy of Wharton’s jelly– derived Mesenchymal Stromal Cells (WJMSC) in graft versus host disease (GVHD). Additional trials explore the infusion of donor derived gamma delta T-cell infusion after haplo-identical stem cell transplant in patients with acute leukemia. The latter strategy seeks to prevent relapse after transplant, while avoiding the risk of untoward GVHD. Further, KUCC translational scientists are developing novel CAR-T constructs to target triple negative breast cancer and glioblastomas. There are currently 23 active clinical trials in KUCC using cellular therapeutics to target both hematologic and solid tumor cancers.

In 2019, KUCC-MCA (Masonic Cancer Alliance) was selected as an NCI Community Oncology Research Program (NCORP) Minority/Underserved Community Site (UG1). The KUCC-MCA Rural NCORP leverages our 45+ year experience as a SWOG-member, 25-year experience providing oncology care via telemedicine, 15-year history of successfully conducting research in rural primary care settings, and 10-year experience opening and running clinical trials within rural healthcare networks. The overarching goal is to enhance capacity for increasing clinical trial accruals and participation of rural populations in cancer control, prevention, treatment, and care delivery clinical trials. In 2020, 183 patients were accrued to NCORP trials, outpacing the annual NCORP goal of 150 accruals. In 2020, we were awarded a Create Access to Targeted Cancer Therapy for Underserved Populations (CATCHUP 2020) grant to expand access to ETCTN trials for rural and underserved populations. KUCC was one of just eight NCI CCSGs to receive the CATCH-UP 2020 administrative supplement, and we were the only institution specifically cited for enrolling rural patients on clinical trials. This congressionally mandated P30 supplement aims to enhance access to targeted cancer therapies for minority/underserved populations. The CATCH-UP 2020 trials were opened at KUCC community sites and MCA (Masonic Cancer Alliance) outreach centers. The average time to activate these trials was 41 days and 55% were enrolled from rural/minority/underserved population/area.

Fostering the growth and further development of the infrastructure to support clinical research is a continued area of emphasis for KUCC to ensure we maximize our impact on the catchment area. The KUCC clinic trial office (CTO) provides comprehensive support services that span the life cycle of cancer clinical trials from concept through manuscript. CTO services are available to all members of the Cancer Center’s research programs.

The CTO provides centralized protocol management and reporting, with strong emphasis on data integrity, protocol compliance, education and training of CTO staff and investigators, and timelines for rapid trial submission and activation. The CTO supports KUCC investigators through the Disease Working Groups (DWG) to develop research portfolios that meet the needs of our catchment area. Enhancements to CTO operations enabled the growth of clinical research, with focus on investigator-initiated trials (IITs), early phase clinical trials and trials for underserved and rural populations. Cancer Center Program Leaders are integrated into CTO committees including the Clinical Research Steering Committee and IIT Steering Committee so that CTO functions best serve the needs of the individual programs and promote translation of KUCC science to the clinic. The CTO expansion and staff specialization has enabled a steady increase in clinical trial accrual. From 2016–2020, there were 13,575 accruals in interventional, observational, and ancillary/correlative studies. Accrual to interventional treatment clinical trials increased by 61% (3,965 accruals in 2016–2020 versus 2,462 in 2011–2015). Accrual to NCTN protocols increased overall by 111% (1,581 accruals in 2016–2020; 750 from 2011–2015). Our achievements led to our recognition as an NCTN “High-Performing Site,” and awards of an NCORP grant and CATCH UP 2020 supplement focused on rural and underserved populations. Our commitment to increase clinical trial outreach to local community sites and the Masonic Cancer Alliance resulted in a 62% increase in accruals (404 accruals in 2016–2020; 506 accruals in 2011–2015). The creation of a dedicated IIT team within the CTO and the IIT Steering Committee resulted in an increase in accruals to interventional treatment IITs by 151% (1,645 accruals in 2016–2020; 656 in 2011–2015).

As a key member of NCI comprehensive cancer centers, the success of clinical research at KUCC demonstrates how critical investigational clinical trials are in the war against cancer.

